# Targeting Macrophage Migration Inhibitory Factor in Acute Pancreatitis and Pancreatic Cancer

**DOI:** 10.3389/fphar.2021.638950

**Published:** 2021-03-11

**Authors:** Yongjian Wen, Wenhao Cai, Jingyu Yang, Xianghui Fu, Lohitha Putha, Qing Xia, John A. Windsor, Anthony R. Phillips, Joel D. A. Tyndall, Dan Du, Tingting Liu, Wei Huang

**Affiliations:** ^1^ Department of Integrated Traditional Chinese and Western Medicine, Sichuan Provincial Pancreatitis Centre and West China-Liverpool Biomedical Research Centre, West China Hospital of Sichuan University, Chengdu, China; ^2^ Surgical and Translational Research Centre, Faculty of Medical and Health Sciences, University of Auckland, Auckland, New Zealand; ^3^ Applied Surgery and Metabolism Laboratory, School of Biological Sciences, University of Auckland, Auckland, New Zealand; ^4^ Liverpool Pancreatitis Research Group, Liverpool University Hospitals NHS Foundation Trust and Institute of Systems, Molecular and Integrative Biology, University of Liverpool, Liverpool, United Kingdom; ^5^ Division of Endocrinology and Metabolism, State Key Laboratory of Biotherapy, West China Hospital, Sichuan University and Collaborative Innovation Center of Biotherapy, Chengdu, China; ^6^ School of Pharmacy, University of Otago, Dunedin, New Zealand; ^7^ West China-Washington Mitochondria and Metabolism Center, West China Hospital, Sichuan University, Chengdu, China

**Keywords:** macrophage migration inhibitory factor, acute inflammatory response, toll-like receptor, inflammasome, acute pancreatitis, pancreatic cancer

## Abstract

Macrophage migration inhibitory factor (MIF) is a pleiotropic cytokine implicated in the pathogenesis of inflammation and cancer. It is produced by various cells and circulating MIF has been identified as a biomarker for a range of diseases. Extracellular MIF mainly binds to the cluster of differentiation 74 (CD74)/CD44 to activate downstream signaling pathways. These in turn activate immune responses, enhance inflammation and can promote cancer cell proliferation and invasion. Extracellular MIF also binds to the C-X-C chemokine receptors cooperating with or without CD74 to activate chemokine response. Intracellular MIF is involved in Toll-like receptor and inflammasome-mediated inflammatory response. Pharmacological inhibition of MIF has been shown to hold great promise in treating inflammatory diseases and cancer, including small molecule MIF inhibitors targeting the tautomerase active site of MIF and antibodies that neutralize MIF. In the current review, we discuss the role of MIF signaling pathways in inflammation and cancer and summarize the recent advances of the role of MIF in experimental and clinical exocrine pancreatic diseases. We expect to provide insights into clinical translation of MIF antagonism as a strategy for treating acute pancreatitis and pancreatic cancer.

## Introduction

Macrophage migration inhibitory factor (MIF) was originally discovered in 1966 as a lymphokine derived from activated T cells during delayed-type hypersensitivity (Bloom and Bennett, 1966; David, 1966), exhibiting inhibition function of macrophage migration. Since being cloned in the early 1990s (Bernhagen et al., 1993; Bernhagen et al., 1994), numerous researchers have investigated its association with disease, multifaceted versatile functions, receptors, and downstream signaling pathways. MIF is now known to have a pivotal role in metabolic (Morrison and Kleemann, 2015), acute inflammatory (Hertelendy et al., 2018), autoimmune (Greven et al., 2010) and infectious diseases (Leaver et al., 2010), and cancers (O’reilly et al., 2016) including colorectal (He et al., 2009), malignant melanoma (Oliveira et al., 2014), lung (Tomiyasu et al., 2002), breast (Tomiyasu et al., 2002), and prostate (Meyer-Siegler et al., 2002) cancers as well as glioblastomas (Munaut et al., 2002).

Acute pancreatitis (AP) is one of the most common gastroenterological diseases with an increasing global incidence and is complicated by considerable comorbidity, mortality, and financial burden (Peery et al., 2019; Petrov and Yadav, 2019). In the course of the disease, injured pancreatic acinar cells secrete inflammatory mediators such as interleukin-6 (IL-6), tumor necrosis factor-alpha (TNF-α), interleukin-1 beta (IL-1β), MIF, chemokines and their ligands that mediate recruitment and infiltration of neutrophils and monocytes at the injury site (Lugea et al., 2017), further aggravating local injury and systemic inflammation (Linkermann et al., 2014). As a result, anti-inflammatory treatment strategies have been tested in AP (Gukovskaya et al., 2017). Despite an enormous amount of pre-clinical research (Habtezion et al., 2019; Lee and Papachristou, 2019; Saluja et al., 2019) and clinical trials (Moggia et al., 2017), no effective targeted pharmacological treatment for AP has been discovered. Therefore, the current treatment of AP is limited to supportive care as well as the management of local and systemic complications (Vege et al., 2018).

AP, chronic pancreatitis, and pancreatic cancer are the common diseases of the exocrine pancreas (Petrov and Yadav, 2019). About 10% of AP patients will develop chronic pancreatitis (Sankaran et al., 2015) and its global incidence is 10 cases per 100,000 general population per year (Xiao et al., 2016). The estimates of incidence and mortality for pancreatic cancer are 8.14 cases and 6.92 deaths per 100,000 persons annually, respectively (Xiao et al., 2016). In China, pancreatic ductal adenocarcinoma (PDAC) is expected to be the second leading cause of cancer-related death by 2030 (Siegel et al., 2019).

Emerging evidence suggests that inflammatory cytokines including MIF, TNF-α, interferon gamma, and transforming growth factor beta are increased in the setting of cancer (Lippitz, 2013). An elevated cytokine concentration profile is associated with reduced survival in pancreatic cancer patients (Babic et al., 2018). There remains no effective pharmacological treatment for PDAC and the prognosis is still extremely poor (Lippitz, 2013; Ryan et al., 2014). There appears to be a compelling role for MIF in pancreatic diseases. Circulating MIF levels are significantly higher in obese or type 2 diabetic populations compared to healthy controls (Morrison and Kleemann, 2015), are significantly elevated in experimental and human AP and correlated with disease severity (Sakai et al., 2003), and are also highly up-regulated in exosomes (Costa-Silva et al., 2015) and in PDAC tissue (Funamizu et al., 2013; Lippitz, 2013; Tan et al., 2014). Small molecular MIF inhibitors, anti-MIF antibodies, and genetic ablation of MIF have all been tested and show protective effects in experimental AP (Sakai et al., 2003; Matsuda et al., 2006; Guo et al., 2018; Li et al., 2019; Zhu et al., 2020) and PDAC (Winner et al., 2007; Denz et al., 2010; Funamizu et al., 2013; Tan et al., 2014; Costa-Silva et al., 2015; Guo et al., 2016; Yang et al., 2016; Wang et al., 2018; Suresh et al., 2019) models.

The aim of this review is to describe what is known about the structure and function of MIF with a particular focus on signaling pathways involved in inflammation and cancer. The role of MIF in AP and PDAC and the potential for MIF targeted treatment strategies are also emphasised.

## Literature Search

A systematic literature search was conducted in Ovid Medline (PubMed), Scopus, Science Citation Index expanded, and Google Scholar to find related articles. The key words were “acute pancreatitis,” “pancreatitis,” “chronic pancreatitis,” or “pancreatic cancer” in combination with “macrophage migration inhibitory factor” or “D-Dopachrome tautomerase.” All studies investigating MIF in experimental and clinical exocrine pancreatic diseases were collated. Reference lists of relevant reviews and other non-primary data sources regarding this context captured by the search strategy were also manually screened. Only publications in English were included. In total, 15 studies investigating MIF in AP and 14 studies in PDAC were summarized in this review. We did not identify original study investigating MIF and chronic pancreatitis.

## Structure and Function of MIF

MIF is a highly conserved protein of 12.5 kDa, with evolutionarily ancient homologues in plants, protozoans, nematodes, and invertebrates (Sparkes et al., 2017). The MIF protein is a 115-amino acid polypeptide that folds to form two antiparallel α-helices that pack against a 4-stranded β-sheet (Trivedi-Parmar and Jorgensen, 2018). On the basis of X-ray crystallography data, the biologically active form of MIF is a homotrimer (Suzuki et al., 1996). MIF is different from other cytokines because its structure contains three evolutionarily stable catalytic sites that are associated with tautomerase and oxidoreductase activities (Rosengren et al., 1996; Sun et al., 1996; Suzuki et al., 1996).

MIF is secreted by the anterior pituitary and immune cells (Calandra et al., 1994) and is ubiquitously stored and expressed in a variety of cells including epithelial, endothelial, mesenchymal, dendritic, and other cell types (Jankauskas et al., 2019). Constitutive release of MIF from cells results in its high concentration in the extracellular space (Lee et al., 2016; Lang et al., 2018). However, the process by which MIF is released is not fully understood. Unlike other cytokines, MIF exists as a pre-formed type in multiple cell sub-populations throughout the body (Calandra et al., 1994; Bacher et al., 1997), and is particularly distributed and expressed in cells of the nervous (Nishibori et al., 1996) and endocrine systems (Calandra and Roger, 2003) that have direct contact with the natural environment, (e.g. lung, skin, and gastrointestinal). MIF production is largely facilitated in response to an array of stimuli including hypoxia, hydrogen peroxide, lipopolysaccharide (LPS), TNF-α, thrombin, and angiotensin II (Jankauskas et al., 2019). Waeber et al. (Waeber et al., 1997) reported that MIF was highly expressed in several insulin-secreting cell lines, colocalized with insulin-containing secretory granules, and was secreted in response to glucose stimulation in a time- and concentration-dependent manner. Immunoneutralization of MIF by anti-MIF IgG reduced the first and second phase of the glucose-induced insulin secretion response by 39 and 31%, respectively. Whether pancreatic acinar cells produce and release MIF remains to be determined.

## Pathophysiologic Role of MIF

MIF circulates normally at levels from 2 ng/ml to 6 ng/ml, following a circadian rhythm that correlates with plasma cortisol under physiological conditions (Petrovsky et al., 2003). This is of particular clinical relevance as low concentration of glucocorticoids induce release of MIF into circulation and in turn, circulating MIF overrides glucocorticoid-mediated inhibition of cytokine secretion, and has been shown to fully abolish the protective effect of glucocorticoids in a lethal model of endotoxin-induced inflammation (Calandra et al., 1995; Bloom et al., 2016a). In the case of acute inflammatory diseases, MIF has been demonstrated to be implicated in the pathogenesis of glomerulonephritis, acute lung injury, sepsis, and AP, and its elevation is closely associated with disease severity or progression (Harris et al., 2019). In addition to its cytokine activity, mammalian MIF also harbors diverse catalytic functions. In this regard, tautomerase activity is the most widely studied function, exhibiting the ability to catalyze tautomerization of phenylpyruvate, *p*-hydroxyphenylpyruvate, and D-dopachrome (Rosengren et al., 1996; Rosengren et al., 1997). Moreover, MIF interferes in cell cycle regulation by negatively interacting with c-Jun activation binding protein-1 (JAB1, also referred to the fifth component of the constitutive photomorphogenic-9 signalosome, CSN5)-dependent pathways, resulting in degradation of cyclin-dependent kinase inhibitor p27^Kip1^ and cell cycle progression (Kleemann et al., 2000).

## D-Dopachrome Tautomerase

D-dopachrome tautomerase (D-DT, also referred to MIF-2) comprises 117 amino acids with a molecular weight of 13 kDa (Zhang et al., 1995; Nishihira et al., 1998). It has a highly homologous tertiary structure and similar biological properties to MIF (Sugimoto et al., 1999; Merk et al., 2011). Whereas plasma D-DT and MIF circulate in similar concentrations under basal or pathological conditions, LPS-treated macrophages release 20-fold more MIF than D-DT, indicating D-DT derived from nonmacrophage sources prominently contribute to plasma D-DT expressions *in vivo* (Merk et al., 2011). The differences and coincidences between D-DT and MIF have been reviewed by Illescas et al. (Illescas et al., 2020). Regardless of the similarities, D-DT seems to play different or even opposed role from MIF under some circumstances. For example, D-DT lacks both the CXXC redox motif and pseudo (E)LR motifs present on MIF, while the former one is important in sensing redox signals and the latter one is essential for its chemokine function (Esumi et al., 1998; Bernhagen et al., 2007; Merk et al., 2011). In adipose tissues, D-DT and MIF are differentially expressed and have distinct roles in adipogenesis. While D-DT is negatively correlated with obesity and reverses glucose intolerance, MIF is positively correlated with obesity and insulin resistance (Kim et al., 2015). D-DT also binds to JAB1 and the interaction affinity between JAB1 and D-DT is comparable to that observed between JAB1 and MIF (Merk et al., 2011).

## MIF Signalling Pathways in Inflammation and Cancer

**FIGURE 1:**
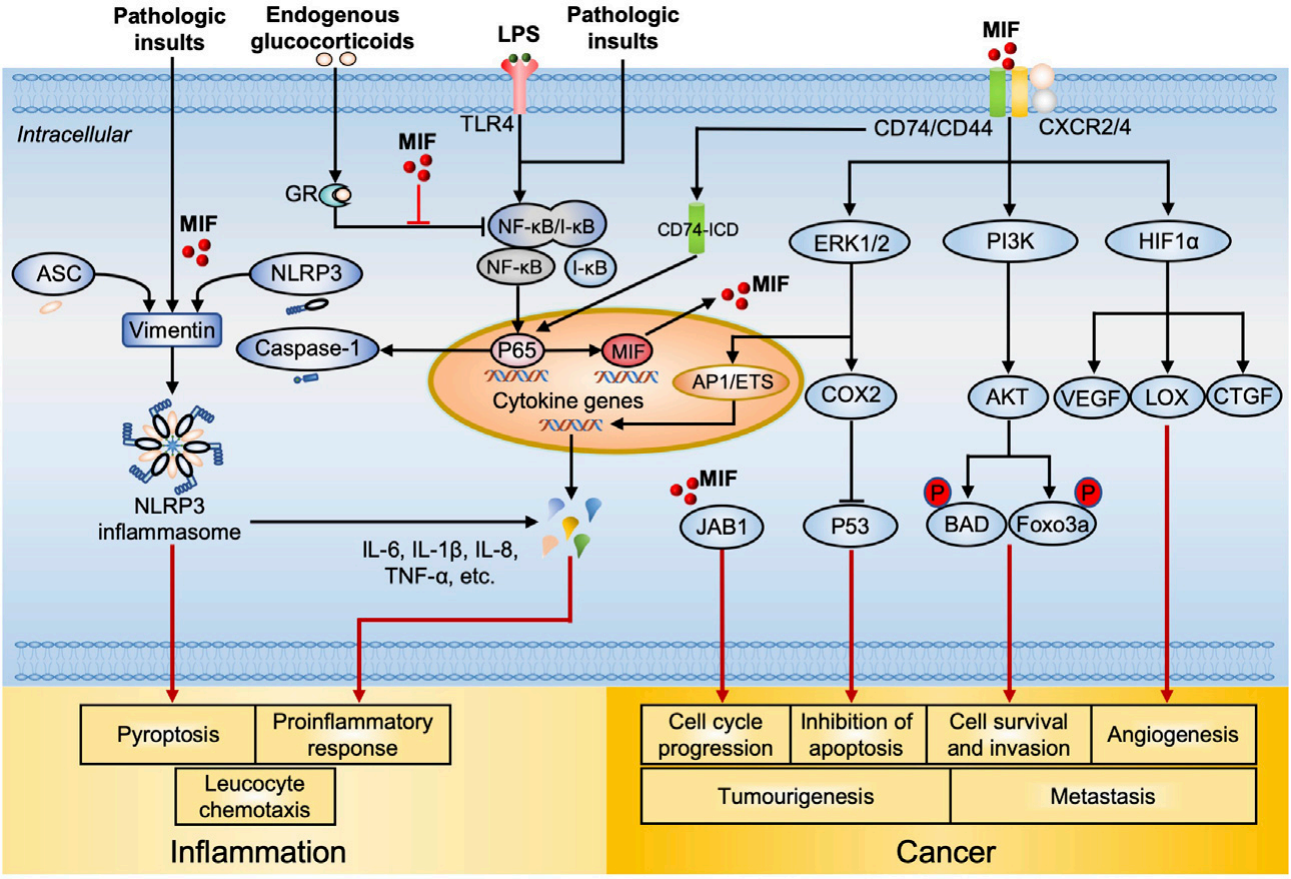
MIF-mediated signal transduction and regulation in inflammation and cancer. Intracellularly, MIF overrides glucocorticoids induced suppression of NF-ĸB synthesis and nuclear translocation, leading to increased cytokine production, (e.g. IL-6 and TNF-α). MIF also facilitates NLRP3 inflammasome assembly through interacting with ASC, vimentin and NLRP3, resulting in pyroptosis and elevated production of IL-1β and IL-18. MIF upregulates the expression of TLR4 by facilitating the transcription of NF-ĸB, which allows rapid recognition of LPS by TLR4, promoting the production of cytokines (including MIF). Intracellular MIF also binds to JAB1, resulting in tumor cell cycle progression. After MIF is released, extracellular MIF binds to the transmembrane receptor complex CD74 and CD44, resulting in the subsequent phosphorylation of ERK/MAPK and PI3K/AKT. ERK1/2 activates transcription elements AP1/ETS, which leads to expression of pro-inflammatory cytokines. On the other side, the activation of ERK/COX2 inhibits p53-dependent apoptosis, promoting tumor cell proliferation. PI3K/AKT activation phosphorylated Bcl-2 family member protein BAD and Foxo transcription factor Foxo3a, enhancing cancer cell survival and invasion. During hypoxia, the binding of MIF to CD74 contributes to HIF1α activation and stabilization, which then upregulates the expression of angiogenic growth factors including VEGF, LOX, and CTGF that consequently promote angiogenesis. Meanwhile, CD74/CD44 receptor complex releases CD74 intracellular domain (CD74-ICD), it translocates into the nucleus and increases NF-ĸB activation, leading to increased caspase-1 activation and NLRP3 production. Extracellular MIF also binds to G-protein-coupled chemokine receptors CXCR2, CXCR4, and CXCR7 individually or dependent on CD74 to form MIF/CXCR2 and MIF/CXCR4 complex, activating leucocyte chemotaxis. Abbreviations: NF-ĸB, nuclear factor kappa B; IL, interleukin; TNF, tumor necrosis factor; NLRP3, NLR Family Pyrin Domain Containing three; TLR4, toll-like receptor four; LPS, lipopolysaccharide; CD74, Cluster of Differentiation 74; ERK, extracellular signal-regulated kinase; MAPK, mitogen-activated protein kinase; PI3K, phosphoinositide 3-kinase; AP1, activator protein 1; COX2, cyclooxygenase-2; BAD, Bcl-2 agonist of cell death; FoxO3a, forkhead box O3a; HIF1α, hypoxia inducible factor 1 alpha; VEGF, vascular endothelial growth factor; LOX, lysyl oxidase; CTGF, connective tissue growth factor; CXCR, C-X-C chemokine receptor.

The MIF-related key signaling pathways in inflammation and cancer are delineated in Figure 1.

### MIF Receptor-Mediated Signaling Pathways

When extracellular MIF binds to its primary receptor cluster of differentiation 74 (CD74) (Leng et al., 2003) on the cell membrane, co-receptors including CD44 or C-X-C chemokine receptors (CXCRs; CXCR2, CXCR4, and CXCR7) (Bernhagen et al., 2007; Alampour-Rajabi et al., 2015) are also required to activate downstream signaling pathways (Shi et al., 2006). Once the CD74/CD44 complex is activated by MIF through the proto-oncogene tyrosine-protein kinase (SRC) (Leng et al., 2003), mitogen-activated protein kinase (MAPK) family members such as the extracellular signal-related kinase 1/2 (ERK1/2), phosphoinositide 3-kinase (PI3K), and protein kinase B (PKB, also known as AKT) are phosphorylated and subsequently activated (Leng et al., 2003; Shi et al., 2006; Lue et al., 2007; Gore et al., 2008). Sustained ERK1/2 activation promotes cancer cell invasion and inhibits cell death (Mitchell et al., 1999). AKT activation leads to phosphorylation of the proapoptotic proteins including Bcl-2 agonist of cell death (BAD) and transcription factor forkhead box O-3a (FoxO3a), promoting cancer cell survival (Lue et al., 2007). MIF-induced cyclo-oxygenase-2 (COX-2)/prostaglandin E2 (PGE-2) activation enhances tumor growth, cancer cell viability, and metastasis. MIF downregulates tumor suppressor protein p53, leading to inhibition of p53-dependent apoptosis, accumulation of mutation, and proliferation of cancer cells (Hudson et al., 1999; Mitchell et al., 2002). ERK1/2 and PI3K/AKT also activate transcription factors including nuclear factor-kappa B (NF-κB) and activator protein-1 (AP1), which result in the release of pro-inflammatory cytokines such as IL-6, IL-8, IL-10, and TNF-α. IL-6 and IL-8 also exhibit pro-tumourigenic functions including promotion of tumor formation by enhancing proliferation, reducing apoptosis, and promoting invasiveness (Taniguchi and Karin, 2014; Johnson et al., 2018). Besides, MIF inhibits p53-mediated apoptosis in macrophage with the induction of increased cytoplasmic phospholipase A2 (PLA2), arachidonic acid, COX2 and PGE2, which sustains the macrophage pro-inflammatory function (Mitchell. et al., 1999; Mitchell et al., 2002). MIF production is upregulated in hypoxic conditions associated with tumor development and progression (Winner et al., 2007). During hypoxia, MIF binding to CD74 contributes to hypoxia inducible factor 1 alpha (HIF1α) activation and stabilization, which then upregulates the expression of angiogenic growth factors including vascular endothelial growth factor (VEGF), lysyl oxidase (LOX), and connective tissue growth factor (CTGF) (Coleman et al., 2008; Oda et al., 2008; Xu et al., 2008; O’reilly et al., 2016). Therefore, MIF supports tumor growth through significantly enhancing angiogenesis. These pathways provide mechanistic bases to explain the role of MIF in the action of pro-inflammatory effects and cancer progression.

MIF can individually bind to CD74 or CXCR2/4 independent of whether the other receptor type is co-expressed (Bernhagen et al., 2007; Schwartz et al., 2009). Extracellular MIF triggers mononuclear (monocytes, neutrophils, and T cells) cell chemotaxis via MIF/CXCR2 and MIF/CXCR4. Monocyte arrest elicited by MIF depends on CD74 and the complex formed by MIF/CXCR2 or MIF/CXCR4 in the context of inflammation and atherosclerosis (Bernhagen et al., 2007). This effect can be suppressed by either a MIF genetic deficiency or antibodies to MIF, CXCR2 or CD74 (Bernhagen et al., 2007). Moreover, MIF can directly interact with CXCR7 (Alampour-Rajabi et al., 2015), resulting in MIF/CXCR7-mediated functional responses that include promotion of human CXCR7 internalization, activation of MIF-mediated ERK1/2 zeta-chain associated protein kinase-70 signaling pathway as well as B cell chemotaxis. It is not completely elucidated whether CD44 is also involved in the receptor complexes of CD74 with the CXCRs. Therefore, tissue responsiveness to MIF depends on its specific expression of MIF receptors and co-receptors, (i.e. CD44 and CXCRs).

### Intracellular MIF-Mediated Signaling Pathways

In addition to its extracellular activities, it is reported that endocytic MIF can transcriptionally and post-transcriptionally override the immunosuppressive effects of glucocorticoids (Calandra and Roger, 2003). Other studies (Roger et al., 2001; Galvão et al., 2016; Lang et al., 2018; Shin. et al., 2019) published in the past few years collectively support a regulatory effect of endogenous MIF on Toll-like receptor 4 (TLR4) and the NLR family pyrin domain containing 3 (NLRP3) inflammasome signaling pathways. These are thought to play a critical role in AP for developing further pancreatic injury and systemic inflammation (Sharif et al., 2009; Sendler et al., 2020). In response to the stimulation by LPS and Gram-negative bacteria (canonical TLR4 activators), the MIF-deficient macrophages were found to be hyposensitive (reduced production of pro-inflammatory cytokines, e.g., TNF-α and IL-6) (Roger. et al., 2001), highlighting a role for MIF in modulation of TLR4 downstream signaling pathways.

MIF as a regulator of the NLRP3-mediated inflammatory response has been described in recent studies (Lang et al., 2018; Shin. et al., 2019). One study showed that MIF colocalized with ASC (apoptosis-associated speck-like protein containing a CARD), vimentin and NLRP3, potentially modulating the interaction between NLRP3 and vimentin to facilitate the NLRP3 inflammasome assembly, leading to acceleration of downstream cytokine release and pyroptosis (Lang et al., 2018). Accordingly, depletion or inhibition of MIF in macrophages and dendritic cells resulted in the inhibition of IL-1α, IL-1β, and IL-18 release in response to NLRP3-activating stimuli. Another study illustrated the link between MIF and NLRP3 activation in human peripheral blood monocytes using U1 small nuclear ribonucleoprotein immune complex, a NLRP3 inflammasome activator (Shin et al., 2012; Shin. et al., 2019), which can stimulate MIF and IL-1β production in human monocytes. MIF levels were increased in synovial fluid and positively associated with IL-1β in a murine acute gout model and human patients (Galvão et al., 2016). Taken together, these studies highlight the role of MIF in modulating activation of downstream events through TLR4/NLRP3 signaling pathways.

Moreover, intracellular MIF binds to JAB1 and results in tumor cell cycle progression and proliferation. MIF-JAB1 interaction also stabilize HIF1α by preventing its hydroxylation (Winner et al., 2007), resulting in increased expression of pro-angiogenic factors such as VEGF and IL-8 (Oda et al., 2008). It subsequently promotes tumor angiogenesis.

## MIF and Exocrine Pancreatic Diseases

### MIF and Experimental AP

Results from studies investigating MIF and experimental AP are summarized in Table 1. Investigation of MIF in AP began in 2003, when Sakai and colleagues demonstrated a functional role for MIF in experimental models of AP induced by taurocholic acid (TCA-AP), caerulein (CER-AP), and choline-deficient, ethionine-supplemented diet (CDE-AP) (Sakai et al., 2003). In isolated peritoneal macrophages from ascitic fluid of TCA-AP rats, co-incubation with anti-MIF antibodies significantly inhibited IL-8 production (Sakai et al., 2003). MIF levels of serum, ascitic fluid, and lung tissue, but not pancreas or liver, were significantly increased in the TCA-AP in rats. Similarly, MIF levels in the lung were also significantly increased in the CDE-AP in mice. As for CER-AP in rats, the MIF levels were only markedly elevated in pancreatic ascites and thus peritoneal macrophages were considered to be the cellular sources of ascitic MIF. In 2006, Matsuda *et al.* (Matsuda et al., 2006) reported that plasma and lung MIF levels were increased by 7-fold and 4.7-fold, respectively, in a severe AP model induced by three injections of caerulein and a low dose of LPS in mice (CER/LPS-AP). The MIF levels in pancreatic tissue and serum were increased in l-arginine-induced AP (ARG-AP) in mice (Ohkawara et al., 2017) and their expression was up-regulated in the intrahepatic bile duct cells in a sodium taurocholate-induced AP (STC-AP) in rats (Wang et al., 2019).

**TABLE 1:** Experimental studies of MIF in acute pancreatitis.

Model	Species	Regimen	Key findings	Refs
Cerulein (6 × 50 μg/kg/h; i.p.)	Wistar rats, male	NA	MIF levels were significantly increased in ascitic fluid but not in serum in AP model	Sakai et al. (2003)
Cerulein (3 × 20 μg/kg/h; s.c.) + LPS (4 mg/kg; i.v., after last cerulein injection)	*Mif* ^−/-^ and wild type BALB/c mice, male	Anti-PAR-2 Ab (100 μg/animal) or anti-MIF Ab (20 mg/animal), i.v., immediately before first cerulein injection	1) acute lung injury was less severe in *Mif* ^*−/−*^ mice of AP complicated by endotoxemia; 2) Anti-MIF Ab or anti-PAR-2 Ab suppressed the AP-induced elevation of lung TLR4 protein expression	Matsuda et al. (2006)
CDE diet (for 48 h)	CD-1 mice, female	Anti-MIF Ab (10 mg/kg) or control rabbit IgG, i.p., immediately after the onset of CDE diet, repeated every 12 h	1) MIF expression was increased in lung of AP model; 2) Anti-MIF Ab improved the survival rate from 16 to 37% in AP mice	Sakai et al. (2003)
l-arginine (2 × 2.5 g/kg; i.p., 1 h interval)	Wistar rats	Glucocorticoid agonist (methylprednisolone; 30 mg/kg) or antagonist (RU-38486; 5 mg/kg), s.c., before disease induction	Antagonist treatment led to significantly higher MIF production at 8 and 12 h after AP induction compared with the agonist-treated or non-treated group	Paszt et al. (2008)
l-arginine (2 × 5 g/kg; i.p., 1 h interval)	C57BL/6 mice, male	Chlorogenic acid (20 or 40 mg/kg; i.p., 1 h before AP induction)	Chlorogenic acid suppressed AP-induced increase of MIF levels in serum and pancreatic tissue	Ohkawara et al. (2017)
l-arginine (2 × 4 g/kg; i.p., 1 h interval)	*Mif* ^−/-^ and WT C57BL/6 mice	ISO-1 (3.5 mg/kg; i.p. 30 min before first l-arginine induction)	1) pancreatic NF-κB p65, IL-1β, and TNF-α, serum IL-1β and TNF-α levels, and multiple organ injury were significantly reduced in *Mif* ^−/-^ mice with AP; 2) ISO-1 markedly reduced severity of AP in wild type mice	Zhu et al. (2020)
TCA (5%, 0.2 ml/min; PD injection)	Wistar rats, male	Anti-MIF Ab (16 mg/kg) or nonspecific rabbit IgG (control) was given 1 h before, immediately after, or 1 h after induction, i.p	1) MIF levels were increased in serum (peak at 9 h: 197 ± 9 ng/ml), ascitic fluid and lung, but not in pancreas or liver in AP model; 2) Anti-MIF Ab reduced lung TNF-α levels and improved survival rate (88 vs. 44%, given 1 h before; 92 vs. 33%, given immediately; 61 vs. 39%, given 1 h after induction) of AP rats	Sakai et al. (2003)
STC (5%, 0.6 ml/kg; PD infusion)	Sprague–Dawley rats, pregnant female	ISO-1 (3.5 mg/kg; i.p., 30 min before STC infusion)	1) MIF expression in fetal liver was elevated in AP which was reduced by ISO-1 treatment; 2) ISO-1 markedly reduced pancreatic and liver histopathological scores, inhibited activation of myeloperoxidase, NF-κB, IL-1β, TNF-α, and HMGB1 in fetal liver in AP rats	Guo et al. (2018)
STC (5%, 1 ml/kg; PD infusion)	Wistar rats, pregnant female	ISO-1 (3.5 mg/kg; i.p., 30 min before STC infusion)	1) ISO-1 alleviated pathological injury of pancreas and lung, attenuated serum levels of IL-1β, IL-6, and TNF-α, inhibited activation of lung p38 MAPK and NF-κB in AP rats; 2) ISO-1 reduced MIF expression, increased expression of p38 MAPK, p-p38, NF-κB, as well as TNF-α and IL-1β levels of fetal kidney tissue in AP rats	Zhou et al. (2018); Li et al. (2019)
STC (5%, 1 ml/kg; PD infusion)	Wistar rats	Ginkgo biloba extract (20 mg/kg; s.c., twice a day pre-operation for 2 days, then given once at the end of the operation)	1) AP resulted in a significant up-regulation expression of MIF and TNF-α proteins in alveolar macrophage; 2) ginkgo biloba extract down-regulated expression of TNF-α (6 h, *p* < 0.001; 12 h, *p* < 0.001) and MIF (6 h, *p* = 0.095; 12 h, *p* < 0.001) in alveolar macrophage compared with AP groups	Xu et al. (2014)
STC (5%, 1 ml/kg; PD infusion)	Wistar rats, male	NA	The expression of MIF mRNA and protein was significantly upregulated in intrahepatic bile duct cells in AP rats	Wang et al. (2019)

MIF, macrophage migration inhibitory factor; TCA, taurocholic acid; PD, pancreaticobiliary duct infusion; Ab, antibody; i. p., intraperitoneal; TNF-α, tumor necrosis factor-alpha; NA, not available; CDE, choline deficient ethionine-supplemented; LPS, lipopolysaccharide; i. v., intravenous; PAR-2, protease activated rec eptor-2; TLR, toll-like receptor; s. c., subcutaneous; STC, sodium taurocholate; ISO-1, (S,R)3-(4-hydroxyphenyl)-4,5-dihydro-5-isoxazole acetic acid methyl ester; NF-κB, nuclear factor kappa B; IL, interleukin; HMGB, high mobility group box; MAPK, mitogen-activated protein kinase.

Compared with wild type littermates, *Mif* ^−/−^ mice reduced pancreatic and serum pro-inflammatory indices as well as severity in ARG-AP (Zhu et al., 2020) and acute lung injury in CER/LPS-AP (Matsuda et al., 2006). Pre-treatment with anti-MIF antibody decreased lung TNF-α levels in the TCA-AP (Sakai et al., 2003) and suppressed the AP-induced elevation of lung TLR4 expression in CER/LPS-AP (Matsuda et al., 2006). Furthermore, anti-MIF antibody significantly reduced lethality in TCA-AP (Sakai et al., 2003), CDE-AP (Sakai et al., 2003), and CER/LPS-AP (Matsuda et al., 2006). MIF specific inhibitor ISO-1 ((S, R)3-(4-hydroxyphenyl)-4, 5-dihydro-5-isoxazole acetic acid methyl ester) significantly mitigated pancreatic (Zhou et al., 2018), lung (Zhou et al., 2018), liver (Guo et al., 2018), and kidney (Li et al., 2019) injury with simultaneous reduction of MIF and a spectrum of pro-inflammatory mediators in STC-AP in pregnant rats. In addition, administration of anti-protease-activated receptor-2 antibody (CER/LPS-AP) (Matsuda et al., 2006), glucocorticoid agonist (ARG-AP) (Paszt et al., 2008), chlorogenic acid (ARG-AP) (Ohkawara et al., 2017), and ginkgo biloba extract (STC-AP) (Xu et al., 2014) also reduced the severity of AP and circulating, pancreatic, or alveolar macrophage MIF levels in experimental models.

### MIF and Human AP

Clinical studies of MIF and AP are displayed in Table 2. Clinical studies (Sakai et al., 2003; Makhija et al., 2007; Rahman et al., 2007; Dambrauskas et al., 2010; Deng et al., 2017) that measured circulating MIF levels collectively and consistently demonstrated that the admission circulating MIF levels were proportionally associated with severity of AP patients. Sakai *et al.* (Sakai et al., 2003) determined serum MIF levels of 28 patients with mild AP and 18 with severe AP within 72 h disease onset in parallel with 12 healthy individuals, revealing that severe AP were associated with markedly higher serum MIF levels compared to mild AP and healthy controls (both *p* < 0.01). But there was no statistical difference between mild AP and controls. Similar results were observed in the study conducted by Rahman *et al.* (Rahman et al., 2007), although the serum samples were collected earlier at 24 h after AP onset. In addition, serum MIF levels were significantly raised in patients who developed pancreatic necrosis or multiple organ failure, indicating MIF could act as a sensitive biomarker to predict local and systemic complications for AP. Another study (Dambrauskas et al., 2010) comprising 108 AP patients (60 mild, 48 severe) also found that serum MIF levels within 72 h disease onset could be used as discriminator of severe and necrotizing AP. A study performed by our group (Deng et al., 2017) identified MIF as an early predictor for discriminating AP patients who had persistent organ failure (n = 20) from those without (n = 50) and healthy controls (n = 10) with an area under the receiver operating characteristic curve (AUC) of 0.90 (95% confidence interval [CI], 0.81–0.96). A most recent study (Shen et al., 2021) demonstrated that MIF at a cut-off value of 2.3 ng/ml has the best discriminative power (AUC, 0.950; 95% CI, 0.914–0.987) for predicting severe AP which was higher than Acute Physiology and Chronic Health Evaluation II (AUC, 0.899), Bedside Index for Severity in Acute Pancreatitis (AUC, 0.886), and serum IL-6 (AUC, 0.826). Therefore, MIF could be used as a potential early severity predictor in patients with AP and this needs further validation.

**TABLE 2:** Patient studies of MIF in acute pancreatitis.

Patient population	Severity definition	Blood sampling time from pain onset	Key findings	Refs
Healthy controls (n = 12). Mild AP (n = 28). Severe AP (n = 18)	OAC	<72 h	Serum MIF levels were significantly higher in severe AP (median 45, range [20–112] ng/ml) compared with mild AP (26 [1–70] ng/ml) or healthy controls (18 [11–34] ng/ml) (both *p* < 0.01)	Sakai et al. (2003)
Healthy controls (n = 10). Mild AP (n = 45). Severe AP (n = 19)	OAC	24 h	1) serum MIF levels were raised in severe AP (median 58, range [13–181] ng/ml), multiple organ failure, or pancreatic necrosis compared with mild AP (20 [5–80] ng/ml) or healthy controls (18 [12–57] ng/ml) (all *p* < 0.01); 2) serum MIF levels significantly correlated with serum 24-h CRP (*r* = 0.36, *p* = 0.02), peak CRP (*r* = 0.36, *p* = 0.003), and 48-h APACHE II score (*r* = 0.29, *p* = 0.03). 3) 24-h serum MIF was superior to sCD14 and sCD163 in predicting severity (AUC, 0.84), multiple organ failure (AUC, 0.80), and pancreatic necrosis (AUC, 0.86) (all *p* < 0.001)	Rahman et al. (2007)
Healthy controls (n = 197). Mild AP (n = 116). Severe (n = 48)	OAC	NA	1) AP vs. controls for distribution of MIF-173 gene genotype (*p* = 0.046); 2) AP vs. controls for distribution of MIF-794 microsatellite genotypes (*p* = 0.367) and alleles (*p* = 0.342)	Makhija et al. (2007)
Healthy controls (n = 18). Mild AP (n = 60). Severe (n = 48)	APACHE II > 7, imrie-glasgow > 2, or MODS >2	<72 h	1) peripheral leukocyte mRNA levels of MIF in AP patients were 6.5-fold higher than healthy controls; serum MIF levels in AP patients were 10.3-fold higher than controls; 2) prognostic utility of serum MIF: Severity: Cutoff (>1,186 pg/ml), AUC (0.71), Se = 0.47, Sp = 0.93, *p* < 0.01. Necrosis: Cutoff (>2,707 pg/ml), AUC (0.55), Se = 0.23, Sp = 0.95, *p* = 0.47. Death: Cutoff (>633 pg/ml), AUC (0.84), Se = 1.00, Sp = 0.61, *p* < 0.01	Dambrauskas et al. (2010)
Healthy controls (n = 10). Mild AP (n = 20). Moderately severe AP (n = 30). Severe AP (n = 20)	RAC	<72 h	1) plasma MIF levels were elevated in AP patients and were associated with disease severity (control: IQR 296, [57–557] pg/ml; mild: 438 [143–1,453] pg/ml; moderately severe: 717 [201–2,631] pg/ml; severe: 2,984 [74–44,786] pg/ml); 2) prognostic utility of plasma MIF in discriminating severe AP from non-severe AP and healthy controls: Cutoff (>1,520 pg/ml), AUC (0.90), Se = 0.75, Sp = 0.98, *p* < 0.001	Deng et al. (2017)
Healthy controls (n = 10). Mild AP (n = 52). Moderate severe AP (n = 65). Severe AP (n = 26)	RAC	<48 h	1) plasma MIF levels were elevated in non-severe AP (1.68 ± 2.04 ng/ml) and severe AP (6.04 ± 4.05 ng/ml) than healthy controls (0.51 ± 0.23 ng/ml); 2) prognostic utility of plasma MIF in discriminating severe AP from non-severe AP and healthy controls: Cut-off (> 2.30 ng/ml), AUC (0.950), Se = 0.962, Sp = 0.803	Shen et al. (2021)

MIF, macrophage migration inhibitory factor; AP, acute pancreatitis; OAC, original Atlanta classification; CRP, C-reactive protein; APACHE II, Acute Physiology and Chronic Health Evaluation II; AUC, area under the receiver operating characteristic curve; RAC, revised Atlanta classification; Se, sensitivity; Sp, specificity.

Previous studies described the ‘G’ to ‘C’ single nucleotide polymorphism (at −173 position) of MIF in patients with systemic-onset juvenile idiopathic arthritis (Donn et al., 2001) and sarcoidosis (Amoli et al., 2002), and a CATT repeat microsatellite (at -794 position) to be associated with lower disease severity in rheumatoid arthritis (Baugh et al., 2002). Accordingly, Makhija et al. (Makhija et al., 2007) compared the MIF gene polymorphism of a United Kingdom cohort of 164 AP patients with 197 healthy controls. It is shown that the distribution of MIF-173 genotype was significantly different between the two groups (*p* = 0.046), whilst there was no difference regarding the distribution of MIF-794 microsatellite genotypes and alleles. However, these findings warrant confirmation from a larger population.

### MIF and Experimental Pancreatic Cancer

The *in vitro* and *in vivo* studies of MIF and pancreatic cancer are outlined in Tables 3, 4. The levels of MIF expression vary in pancreatic cancer cell lines with high over-expression in PT-45, CFPAC-1, PaCa2, and Capan-1 cells (Ct < 6.5) (Denz et al., 2010). MIF over-expression is associated with reduced E-cadherin expression and increased vimentin expression, indicative of epithelial-to-mesenchymal transition characteristics thus enhanced invasiveness in pancreatic cancer cell lines (Funamizu et al., 2013). Besides, MIF over-expression is also associated with increased proliferation and reduced sensitivity to gemcitabine (Funamizu et al., 2013; Yang et al., 2016). Activation of PI3K/Akt (Denz et al., 2010; Guo et al., 2016; Yang et al., 2016; Wang et al., 2018) and ERK (Guo et al., 2016; Wang et al., 2018) mediated signaling pathways were demonstrated to be involved in the process. MIF knockdown by siRNA attenuated proliferation and invasion along with increased apoptosis in pancreatic cancer cell lines via upregulation of p53 and downregulation of ERK1/2 and AKT phosphorylation (Denz et al., 2010; Guo et al., 2016; Wang et al., 2018). MIF inhibitor (4-iodo-6-phenylpyrimidine; 4-IPP) reduced proliferation and colony formation in PANC-1 cells (Guo et al., 2016).

**TABLE 3:** *In vitro* studies of MIF in pancreatic cancer.

Cell types	Interventions	Key findings	Refs
MIA PaCa-2, PANC-1	MIF knockdown/knockout	1) hypoxia induced MIF expression and secretion in PC cell lines in a HIF-1α–dependent manner; 2) MIF was required for maximal hypoxia-induced HIF-1α stabilization in PC cell lines; 3) MIF bound to CSN5 in PC cell lines and MIF depletion resulted in a loss of CSN5 binding and stabilization of HIF-1α	Winner et al. (2007)
MIA PaCa-2, AsPC-1, BxPC-3, Capan-1, CFPAC-1, HPAF-II, PANC-1, Colo357, PANC-89, PancTuI, PT-45, PT-64	MIF knockdown	1) qRT-PCR of MIF in PC cell lines showed PT-45, CFPAC-1, PancTuI, and Capan-1 cells displayed the highest MIF expression (Ct < 6.5), Panc-89 and Panc-1 showed low expression levels (Ct > 8). AsPC-1, Colo357, BxPC-3, PT-64, HPAF-II, and MIA PaCa-2 showed an average expression of MIF (7 < Ct < 8); 2) MIF knockdown cells showed decreased proliferation and viability and increased apoptosis; 3) MIF knockdown downregulated total Akt expression but increased phosphitylation at the Thr308 residue of Akt	Denz et al. (2010)
PANC-1, Capan-2	Overexpression and knockdown of MIF, gemcitabine	1) MIF overexpression decreased E-cadherin and increased vimentin in PC cells, consistent with the features of EMT; 2) MIF overexpressing PC cells showed significantly higher invasive ability and increased proliferation than vector control cells, sensitivity to gemcitabine was reduced	Funamizu et al. (2013)
PANC-1, Capan-2, HPDE6, HIT-T15	Knockin and knockdown of MIF, rMIF, PP1, AZD0530	1) rMIF inhibited insulin secretion of isolated mouse islets and HIT-T15 cells on a dose-dependent pattern; 2) rMIF depressed VDCC Ca^2+^ currents in HIT-T15 cells via regulating Src family tyrosine kinase activity; 3) the regulatory effect of PANC1, Capan-2, and MIF-expressing HPDE6 cells on insulin secretion from islet cells was significantly ameliorated by using PP1 or AZD0530	Tan et al. (2014)
PANC-1, BxPC-3, ASPC-1, HEK293	Knockdown of DDT and MIF, 4-IPP	1) knockdown of MIF and DDT synergistically inhibited ERK1/2 and Akt phosphorylation, increased p53 expression and attenuated proliferation and invasion of PANC-1 cells; 2) 4-IPP reduced PANC-1 proliferation and colony formation	Guo et al. (2016)
PANC-1, CFPAC-1, Capan-2, MIA PaCa-2	LY294002 (PI3K/Akt inhibitor), AZD6244 (MEK inhibitor)	1) MIF-induced increase in miR-301b targets and reduces NR3C2 levels in PANC-1 and Capan-2 cell lines; 2) NR3C2 inhibited proliferation, colony formation, migration, invasion, and enhances sensitivity of PC cell lines to gemcitabine; 3) MIF enhanced PC cell lines invasiveness by targeting NR3C2 through the upregulation of miR-301b; 4) AZD6244 didn’t alter miR-301b or NR3C2 in MIF-overexpressing PC cell lines; 5) treatment of MIF-overexpression PC cells with LY294002 resulted in miR-301b decrease and NR3C2 increase	Yang et al. (2016)
PANC-1, BxPC-3	MIF knockdown	1) knockdown of MIF suppressed proliferation and invasion of PDAC cells; 2) knockdown of MIF inhibited the activation of Akt and ERK, and suppressed the expression of cyclin D1 and MMP-2	Wang et al. (2018)
Hamster PBMCs, HapT1	Recombinant MaMIF, ISO-1, MaMIF knockdown	1) the primary sequence, biochemical properties, and crystal structure of MaMIF showed great similarity with human MIF; 2) recombinant MaMIF induced significantly higher expression of TNF-α, IL-6, and VEGF in hamster PBMCs than non-treated group, ISO-1 suppressed the expression of these factors in PBMCs; 3) recombinant MaMIF showed no effect on the overall growth of HapT1 cells; 4) intracellular MIF knockdown by siRNA or inactivation by ISO-1 reduced overall growth of HapT1 cells	Suresh et al. (2019)

MIF, macrophage migration inhibitory factor; PC, pancreatic cancer; HIF-1α, hypoxia inducible factor-1α; CSN5, COP9 signalosome subunit five; PDAC, pancreatic ductal adenocarcinoma; AUC, area under the receiver operating characteristic curve; EMT, epithelial-to-mesenchymal transition; IHC, immunohistochemistry; PTX3, pentraxin3; rMIF, recombinant MIF; PP1, protein phosphatase one; AZD0530, saracatinib; VDCC, voltage-gated calcium channel; PanIN, pancreatic intraepithelial neoplasias; DDT, D-dopachrome tautomerase; 4-IPP, 4-iodo-6-phenylpyrimidine; ERK, extracellular signal-regulated kinases; oxMIF, oxidative MIF; PI3K, phosphoinositide 3-kinase; MEK, mitogen-activated protein kinase; NR3C2, nuclear receptor subfamily three group C member two; MMP-2, matrix metalloproteinase-2; PBMCs, peripheral blood mononuclear cells; MaMIF, *Mesocricetus auratus* MIF; ISO-1, (S, R)-3-(4-hydroxyphenyl)-4, 5-dihydro-5-isoxazole acetic acid methyl ester; TNF-α, tumor necrosis factor-α; IL-6, interleukin-6; VEGF, vascular endothelial growth factor.

**TABLE 4:** *In vivo* studies of MIF in pancreatic cancer.

Model	Interventions	Key findings	Refs
Orthotopic xenografts (Capan 2 cells)	MIF overexpression	MIF overexpression enhanced primary tumor growth and metastasis	Funamizu et al. (2013)
1) PKCY mouse PC model; 2) intraplenic injection (PAN02 cells)	MIF knockdown	1) levels of MIF in exosomes derived from plasma of PKCY mice at PanIN and PDAC stages were significantly higher than controls; 2) MIF-expressing PAN02 exosomes education induced liver pre-metastatic niche formation and metastasis, was inhibited by MIF knockdown	Costa-Silva et al. (2015)
Subcutaneous injection (PANC-1 cells)	4-IPP	4-IPP treatment reduced tumor formation in mice within 28 days but did not change the expression of MIF or DDT in xenograft tumors, compared with vehicle treatment	Guo et al. (2016)
KPC mouse PC model	MIF knockout	MIF knockout disrupted MIF–miR-301b–NR3C2 axis, enhanced survival, and reduced metastasis in KPC mouse model	Yang et al. (2016)
Subcutaneous injection (HapT1 cells)	Recombinant MaMIF	Recombinant MaMIF significantly enhanced tumor growth in hamster via promoting tumor angiogenesis	Suresh et al. (2019)

MIF, macrophage migration inhibitory factor; PC, pancreatic cancer; 4-IPP, 4-iodo-6-phenylpyrimidine; DDT, D-dopachrome tautomerase; NR3C2, nuclear receptor subfamily three group C member two; MaMIF, *Mesocricetus auratus* MIF.

A recent study (Suresh et al., 2019) found that recombinant MIF treatment significantly enhanced tumor growth via promoting angiogenesis in a hamster pancreatic cancer model induced by subcutaneous inoculation with HapT1 cells. Conversely, MIF knockdown or inhibition (ISO-1) reduced overall cell growth in HapT1 cells.

Intriguingly, MIF has been linked to exosomes in PDAC. Costa-Silva *et al.* (Costa-Silva et al., 2015) demonstrated that MIF levels in plasma exosomes isolated from a mouse pancreatic cancer model were markedly increased compared to non-cancerous controls, even at an early stage of pancreatic intraepithelial neoplasia. The process of MIF-expressing PAN02 exosome “education”, (whereby naive, wild-type mice were injected retro-orbitally every other day for three weeks with 5 μg of PAN02-derived exosomes), induced liver pre-metastatic niche formation in treated mice which was also inhibited by MIF knockdown.

Yang *et al.* (Yang et al., 2016) demonstrated the role of MIF–miR-301b–NR3C2 axis in the pathogenesis of PDAC. Over-expression of MIF induced a marked increase of miR-301b and reduction of NR3C2 levels, resulting in profound proliferation, migration, and invasion of pancreatic cancer cell lines (PANC-1 and Capan-2). While inhibition of miR-301b abolished MIF-induced suppression of NR3C2 *in vitro*, *Mif* ^−/−^ mice were associated with reduced metastasis and improved survival in a pancreatic cancer mouse model (*LSL-Kras* ^*G12D*^ *, LSL-Trp53* ^*R172H/+*^ *, Pdx-1-Cre*) *in vivo*.

### MIF and Human Pancreatic Cancer

Clinical studies of MIF and pancreatic cancer are presented in Table 5 **.** Collectively, these clinical studies have determined the circulating MIF levels (Winner et al., 2007; Fredriksson et al., 2008; Chen et al., 2010; Kondo et al., 2013; Tan et al., 2014; Schinagl et al., 2016) and MIF expression in dissected pancreatic tissue (Cui et al., 2009; Denz et al., 2010; Funamizu et al., 2013; Tan et al., 2014; Guo et al., 2016; Schinagl et al., 2016; Yang et al., 2016; Wang et al., 2018) of PDAC patients and healthy controls. All the studies reported that MIF levels in serum/plasma or pancreatic tissue were significantly higher in PDAC patients than healthy controls or paired non-cancerous tissue with preferable diagnostic utility, except for one study (Fredriksson et al., 2008) in which there was 30% increase in PDAC without significant difference compared with healthy controls (*p* = 0.15). Higher MIF expression in tumor tissue was associated with worse survival of PDAC patients (Funamizu et al., 2013; Wang et al., 2018).

**TABLE 5:** Patient studies of MIF in pancreatic cancer.

Sampling type	Key findings	Refs
Plasma. PC patients (n = 30), healthy controls (n = 10)	Plasma MIF levels in PC (mean 36 ng/ml) was significantly higher than healthy controls (24 ng/ml) (*p* = 0.038)	Winner et al. (2007)
Plasma. PC patients (n = 18), healthy controls (n = 19)	Plasma MIF levels in PC were not significantly different from healthy controls with an average increase of 30% (*p* = 0.15)	Fredriksson et al. (2008)
Plasma. PDAC patients (n = 78)	1) mean plasma MIF levels in PDAC patients was 7,240 pg/ml; 2) plasma PTX3 levels were positively correlated with levels of MIF (*r* = 0.38, *p* = 0.001)	Kondo et al. (2013)
Serum. PC (n = 17), AP (n = 26), CP (n = 26), healthy controls (n = 16)	1) compared with healthy controls, serum MIF levels in PC patients and pancreatitis patients were 10.6-fold and 9.2-fold higher, respectively; 2) the AUC of serum MIF in discriminating PC patients from healthy controls or all controls (including AP, CP, and healthy controls) were 1.00 or 0.78, respectively	Chen et al. (2010)
Pancreatic tissue. DM-PC, CP, and healthy controls. Serum. PC and non-PC patients: New-onset DM-PC (n = 35), PC without DM (n = 35), new-onset T2DM (n = 35), healthy controls (n = 35)	1) MIF expression was significantly higher in DM-PC tissues than CP or PC patients without DM; 2) mean serum MIF levels (ng/ml) were higher in new-onset DM-PC (32), vs. PC without DM (17, *p* < 0.001), long-term DM-PC (20, *p* < 0.001), new-onset T2DM patients (21, *p* < 0.01), and healthy controls (14, *p* < 0.001); 3) diagnostic utility of serum MIF in distinguishing new-onset DM-PC from new-onset T2DM: AUC, 0.85; Se, 86%; Sp, 60%	Tan et al. (2014)
Pancreatic tissue. PDAC patients (n = 11), healthy controls (n = 7)	1) MIF protein expression in PC nests was 2.7-fold higher than normal pancreatic ducts; 2) the AUC of pancreatic MIF levels in discriminating PC from normal pancreas was >0.7 with *p* < 0.001	Cui et al. (2009)
Pancreatic tissue. PDAC patients (n = 11), CP (n = 9), ***Paired tissue.*** PDAC patients (n = 11), healthy controls (unknown)	MIF mRNA expression in pancreatic tissues was higher in PDAC than CP, stromal tissue of PDAC, and normal ductal area	Denz et al. (2010)
Pancreatic tissue. PDAC patients (n = 57)	1) higher MIF expression in tumors was associated with poorer survival independent of tumor stage; 2) IHC showed an increased expression of MIF in cancer cells compared with surrounding non-tumor ductal cells	Funamizu et al. (2013)
Pancreatic tissue. PDAC patients (n = 64)	DDT was over-expressed in PDAC tissues in a pattern positively correlated with that of MIF (*r* = 0.346, *p* = 0.0001)	Guo et al. (2016)
Plasma and pancreatic tissue. PanIN and PDAC patients (n = 40), healthy donors (n = 91)	1) plasma levels of both total MIF and oxMIF were not significantly different between PC patients and healthy donors; 2) in IHC, oxMIF was over-expressed in PanINs and PDAC tissues and was correlated with cancer stage, stronger in later stage tumors; adjacent normal pancreatic tissue did not show oxMIF staining	Schinagl et al. (2016)
Pancreatic tissue. PDAC patients: MIF-high (n = 43), MIF-low (n = 42)	1) the majority of PDAC tumor tissue (75/85) exhibited significantly higher MIF expression than paired noncancerous tissue; 2) higher MIF expression in tumor tissue is associated with poor survival of PDAC patients (*p* = 0.023)	Wang et al. (2018)
Pancreatic tissue. PDAC patients: 1) test cohort (n = 69): MIF-high (n = 35), MIF-low (n = 34); 2) validation cohort-1 (n = 41): MIF-high (n = 21), MIF-low (n = 20); 3) validation cohort-2 (n = 69): MIF-high (n = 35), MIF-low (n = 34)	1) miRNA profiling identified 53 differentially expressed miRNAs in MIF-high vs. MIF-low tumors, a higher expression of miR-301b, miR-15b, miR-10b, miR-93, and miR-590–5p in MIF-high tumors were also associated with poor survival in PDAC cases; 2) MIF expression in the tumor was positively correlated with miR-301b and negatively correlated with NR3C2 expression	Yang et al. (2016)
Exosomes isolated from plasma. PDAC patients: With liver metastasis (n = 18), with no evidence of disease five years post-diagnosis (n = 10), with progression of disease post-diagnosis (n = 12), healthy controls (n = 15)	1) MIF levels in exosomes isolated from PDAC with progression of disease post-diagnosis were significantly higher than PDAC with no evidence of disease 5 years post diagnosis and healthy controls; 2) MIF levels were lower in PDAC patients with liver metastasis that those with progression of disease, without significance	Costa-Silva et al. (2015)

MIF, macrophage migration inhibitory factor; PC, pancreatic cancer; PDAC, pancreatic ductal adenocarcinoma; AUC, area under the receiver operating characteristic curve; AP, acute pancreatitis; CP, chronic pancreatitis; IHC, immunohistochemistry; PTX3, pentraxin3; DM-PC, pancreatic cancer associated diabetes mellitus; T2DM, type-2 diabetes mellitus; Se, sensitivity; Sp, specificity. PanIN, pancreatic intraepithelial neoplasia; DDT, D-dopachrome tautomerase; oxMIF, oxidative MIF.

In addition, Denz *et al.* (Denz et al., 2010) reported that MIF mRNA expression in pancreatic tissue was higher in PDAC than chronic pancreatitis, both were higher than normal controls. Chen et al. (Chen et al., 2010) compared the serum MIF in PDAC patients and controls including AP, chronic pancreatitis and healthy donors, showing that MIF had an area under the curve of receiver operating characteristic of 0.78 in discriminating pancreatic cancer from controls. Tan et al. (Tan et al., 2014) focused on diabetes mellitus-associated pancreatic cancer (DM-PC) and found that MIF expression in pancreatic tissues of DM-PC was markedly higher when comparing to chronic pancreatitis or pancreatic cancer without DM. Similarly, serum MIF levels were also higher in new-onset than long term DM-PC, pancreatic cancer without DM, or new-onset T2DM patients (all *p* < 0.001).

## MIF Targeted Treatment Strategies

**FIGURE 2:**
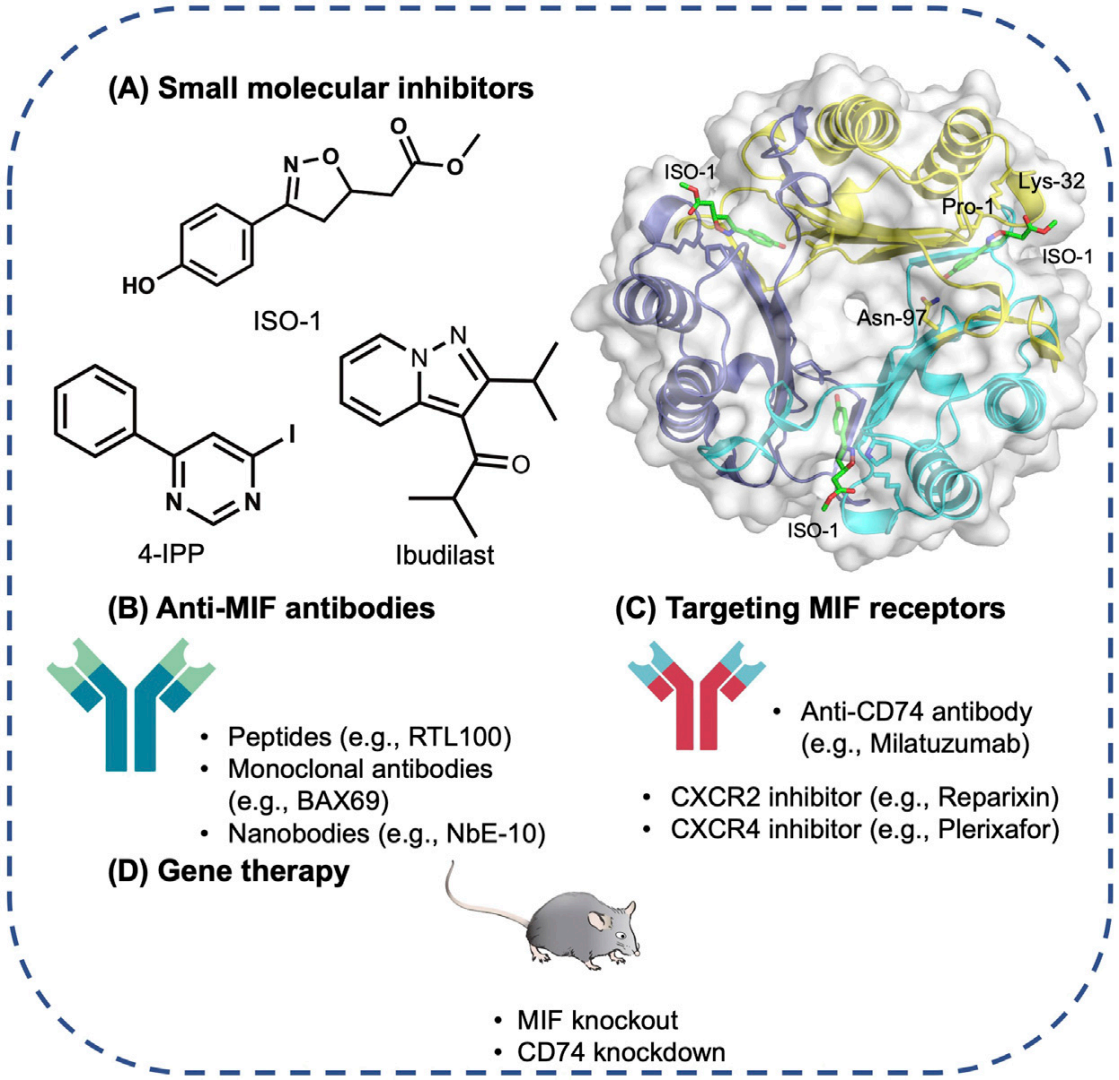
Therapeutic strategies targeting MIF. **(A)** Compounds bind to MIF’s tautomerase active site. For example, ISO-1 binds to the active-site residue Asn-97 of MIF (PDB 1LJT), leading to structural changes that block MIF–CD74 binding; 4-IPP covalently binds to Pro-1 of MIF or DDT, altering their structure and preventing function; and the non-competitive allosteric tautomerase inhibitor Ibudilast. **(B)** Anti-MIF antibodies. **(C)** Drugs targeting MIF receptors. **(D)** Gene therapy. Abbreviations: ISO-1, (S, R)3-(4-hydroxyphenyl)-4, 5-dihydro-5-isoxazole acetic acid methyl ester; CD74, Cluster of Differentiation 74; 4-IPP, 4-iodo-6-phenylpyrimidine; DDT, D-dopachrome tautomerase.

Different MIF antagonism strategies are depicted in Figure 2.

### Small Molecular Inhibitors

Pharmacological inhibition of MIF with small molecule inhibitors have shown promise in the suppression of inflammation in various animals models such as severe sepsis (Calandra et al., 2000), rheumatoid arthritis (Mikulowska et al., 1997), allergic airway inflammation (Amano et al., 2007), colitis (Ohkawara et al., 2002), glomerulonephritis (Brown et al., 2002), and chronic obstructive pulmonary disease (Russell et al., 2016). Development of MIF inhibitors has been comprehensively and elegantly reviewed elsewhere (Xu et al., 2013; Kok et al., 2018; Trivedi-Parmar and Jorgensen, 2018). Small inhibitory agents of MIF have also been widely demonstrated in experimental cancer models, including lung cancer (Mawhinney et al., 2015), bladder cancer (Choudhary et al., 2013), adenoid cystic carcinoma (Liu et al., 2013), melanoma, and colon cancer (Ioanou et al., 2014). Briefly, small-molecule inhibitors of MIF primarily focus on rational structure-based design that target the MIF tautomerase activity and MIF-CD74 binding (Brown et al., 2009; Xu et al., 2013; Dickerhof et al., 2014; Spencer et al., 2015; Trivedi-Parmar and Jorgensen, 2018). The drought of a reliable, consensus-based *in vitro* assays for MIF biological activity has been a significant challenge to the development of small molecule MIF inhibitors. Further work should focus on developing a robust high-throughput clinically relevant MIF bioassay that can be applied for second-pass screening, glucocorticoid override, cellular proliferation, and cytokine release detection to expedite the discovery of efficient small molecular MIF inhibitors (Bloom et al., 2016b). In AP and PDAC, only ISO-1 and 4-IPP have been investigated in experimental animal models so far with limited data available, more endeavors are required to test small molecular MIF inhibitors in AP and PDAC *in vitro* and *in vivo* thus to consolidate the evidence for clinical translation.

### Anti-MIF Antibodies

In AP, two studies (Sakai et al., 2003; Matsuda et al., 2006) have demonstrated the efficacy of anti-MIF antibody in CER/LPS and CDE diet AP models, respectively, revealing a promising therapeutic target. A newly established series of nanobodies (NbE5 and NbE10) have shown to attenuate lethality in *in vivo* septic shock model (Sparkes et al., 2018), is of great interest to be applied in other conditions of inflammatory end-organ damage, such as severe AP. A number of MIF monoclonal antibodies developed by researchers at Baxter have revealed significant anti-MIF activity in human PC3 prostate cancer cell lines and *in vivo* xenograft model (Hussain et al., 2013). *In vitro*, BaxG03, BaxB01, and BaxM159 reduced cell growth and viability by inhibiting ERK1/2 and AKT pathways. The antibodies also inhibited MIF-promoted migration and invasion. *In vivo*, treatment with anti-MIF antibodies reduced tumor growth in a dose-dependent manner. Recently, a phase 1 clinical trial using imalumab (Bax69) in solid tumors and metastatic colorectal adenocarcinoma demonstrated that imalumab has a maximum tolerated dose of 37.5 mg/kg every 2 weeks and a biologically active dose of 10 mg/kg weekly (Mahalingam et al., 2020). Further investigation is warranted to define the role of anti-MIF antibody as a treatment strategy for pancreatic cancer. Of note, the development of antibodies may be mitigated by their short half-life, high costs associated with production, and potential immunogenicity.

### Targeting MIF Receptors

As MIF relies largely on CD74 to regulate the downstream cellular events, treatment targeting CD74 holds great potential to inhibit MIF signaling. A humanized anti-CD74 monoclonal antibody, milatuzumab has shown to significantly prolong the survival duration of multiple myeloma xenograft mice models (Stein et al., 2004; Stein et al., 2009). It has reached phase 1 clinical trial in multiple myeloma (Kaufman et al., 2013) and systemic lupus erythematosus (Wallace et al., 2016), indicating no severe adverse effects. Apart from CD74, inhibitors against CXCL2 (Reparixin) and CXCL4 (Plerixafor) are also of interest for further investigation (Steinberg and Silva, 2010; Goldstein et al., 2020). However, as MIF/CD74 pathway also plays an important role in wound repair by activating pro-survival and proliferative pathways that protects the host during injury (Farr et al., 2020), complete inhibition of CD74 could cause some unpredictable side effects which need precaution.

### MIF-Related Gene Therapy

First of all, MIF knockout animals are long lived with no characteristic health issues, giving the opportunity for the initiation of MIF-related gene therapy (Harper et al., 2010). MIF related gene therapy such as *Mif* gene knockout/knockdown, *DDT* gene knockdown, or *CD74* gene knockdown has exhibited great potential in pre-clinical studies of AP (Matsuda et al., 2006; Zhu et al., 2020) and PDAC ( Winner et al., 2007; Denz et al., 2010; Funamizu et al., 2013; Tan et al., 2014; Costa-Silva et al., 2015; Guo et al., 2016; Yang et al., 2016; Wang et al., 2018; Suresh et al., 2019). Furthermore, it was demonstrated that a xenograft model of head and neck squamous cell carcinoma with MIF knockdown was more sensitive to cisplatin and 5-fluorouracil treatment than control (Kindt et al., 2013). On the other hand, overexpressing MIF in pancreatic cancer cells reduced the sensitivity to gemcitabine (Funamizu et al., 2013). Taken together, it supports that MIF downregulation may potentiate the effect of chemotherapy agents in cancer. There remain ongoing opportunities to develop additional MIF suppression therapies for clinical evaluation.

## Conclusion

In summary, we have comprehensively reviewed the role of MIF in AP and PDAC. It is apparent from the review that investigations of MIF in AP are at a relatively early stage. Up to now, *in vivo* AP studies have measured circulating MIF levels, confirmed MIF expression in target organs, and commenced applying MIF inhibitory drugs for efficacy testing. Whether pancreatic acinar cells express MIF and how MIF contributes to the early acinar cell events in AP, (i.e. calcium overload, mitochondrial dysfunction, oxidative stress, endothelial reticulum stress, and trypsinogen activation) remains unclear. It is notable that anti-MIF antibodies and the MIF inhibitor ISO-1, have shown encouraging potential for improving pancreatic damage and associated organ injury in AP animal models. Clinical studies have identified circulating MIF as a potential biomarker for early prediction of AP severity which needs further validation. Future research is warranted to detail the underlying molecular mechanisms, (i.e. TLR4/NLRP3) of MIF in pancreatic acinar cells and AP. In chronic pancreatitis, the role of MIF remains elusive. In pancreatic cancer, MIF enhances the proliferation and invasion of tumor cells, resulting in increased tumor growth and metastasis *in vivo*. Early studies of MIF knockdown or use of specific inhibitors support MIF as a potential target for PDAC. Future research is required to bring forward a range of promising treatment approaches to clinical evaluation.
